# Addressing technical barriers for reliable, safe removal of fluoride from drinking water using minimally processed bauxite ores

**DOI:** 10.1016/j.deveng.2018.06.002

**Published:** 2018

**Authors:** Heather L. Buckley, Nusrat J. Molla, Katya Cherukumilli, Kathryn S. Boden, Ashok J. Gadgil

**Affiliations:** a Department of Civil Engineering, University of Victoria, Victoria, V8P 5C2 BC, Canada; b Energy Technology Area, Lawrence Berkeley National Laboratory, Berkeley, 94720 CA, USA; c Civil and Environmental Engineering, University of California Berkeley, Berkeley, 94720 CA, USA; d Department of Civil and Environmental Engineering, University of Washington, Seattle, 98195 WA, USA

## Abstract

Throughout the developing world, over 200 million people drink groundwater containing fluoride concentrations surpassing the World Health Organization's maximum recommended contaminant level (WHO-MCL) of 1.5 mg F^−^/L, resulting in adverse health effects ranging from mottled tooth enamel to debilitating skeletal fluorosis.

Existing technologies to remove fluoride from water, such as reverse osmosis and filtration with activated alumina, are expensive and are not accessible for low-income communities. Our group and others have demonstrated that minimally-processed bauxite ores can remove fluoride to safe levels at a fraction of the cost of activated alumina. We report results from testing for some technical challenges that may arise in field deployment of this technology at large scale, particularly in a sufficiently robust manner for application in development contexts. Anticipating possible modes of failure and addressing these challenges in advance in the laboratory is particularly important for technologies for vulnerable communities where the opportunity to re-launch pilot projects is limited and small failures can keep solutions from the people that need them most.

This work addresses three potential technical barriers to reliable removal of fluoride from drinking water with bauxite ore from Visakhapatnam, Andhra Pradesh, India. We evaluate competition from co-occurring ions, adsorption reversibility, and potability of the product water with regards to leaching of undesirable ions during treatment with various adsorbent materials including raw and thermally activated bauxite, and synthetic gibbsite (a simple model system). Under the conditions tested, the presence of phosphate significantly impacts fluoride adsorption capacity on all adsorbents. Sulfate impacts fluoride adsorption on gibbsite, but not on either bauxite adsorbent. Nitrate and silicate (as silicic acid), tested only with gibbsite, do not affect fluoride adsorption capacity. Both thermally activated bauxite and gibbsite show non-reversible adsorption of fluoride at a pH of 6. Raw bauxite leached arsenic and manganese in a TCLP leaching test at levels indicating the need for ongoing monitoring of treated water, but not precluding safe deployment of bauxite as a fluoride remediation technology. Understanding these phenomena is crucial to ensure field deployment over large diverse geographical areas with aquifers varying in groundwater composition, and for ensuring that the appropriate engineering processes are designed for field implementation of this innovation.

## 1 Introduction

Throughout the developing world, over 200 million people drink groundwater containing fluoride concentrations (Edmunds and Smedley, 2013) that exceed the World Health Organization's maximum recommended contaminant level (WHO-MCL) of 1.5 mg F^−^/L. (World Health Organization, 2006; World Health Organization, 2004b) In India alone, over 66 million people risk developing fluorosis due to natural contamination of their drinking water (UNICEF, 1999). In China, where other aspects of quality of life are rapidly improving, as much as 10% of the groundwater-based drinking water supply may contain dangerous levels of naturally occurring fluoride (Wu et al., 2011). The problem is widespread: dissolution of fluoride-rich granitic rocks in groundwater aquifers causes toxic levels of fluoride in arid regions of India, China, the Middle East, the East African Rift Valley, central Argentina, and northern Mexico (Ozsvath, 2008; Jagtap et al., 2012), often in regions without reliable alternative water sources throughout much of the year. This exposes entire communities to devastating health effects, including anemia, reduced cognitive function and dental fluorosis (Brindha and Elango, 2011). At higher concentrations of fluoride in drinking water, skeletal fluorosis leads to irreversible spinal fusion and limb deformation in children, leaving victims severely disabled and often with chronic pain (Khairnar et al., 2015).

As with many public health challenges, poor, rural communities with limited access to healthcare are generally disproportionately affected by the lack of scalable solutions. Existing technologies to remove fluoride from water are both expensive and energy intensive from the perspective of low-income communities, and also have a significant greenhouse gas footprint (Chen and Graedel, 2012a). Many proposed alternative technologies have proven effective in the laboratory (Habuda-Stanic et al., 2014), but scaling these encounters challenges of reliability of water source (e.g., rainwater harvesting) (Mwenge Kahinda et al., 2007), availability of skilled labor for upkeep (e.g. Nalgonda technique) (Jagtap et al., 2012), cultural appropriateness (e.g. bone char in communities with dietary restrictions) (Osterwalder et al., 2014), and myriad challenges with cost, reliability of material sourcing, and wastefulness in water-stressed regions (reverse osmosis is widely used but recovers only about two thirds of the input water) (Mohapatra et al., 2009).

Despite some limitations, aluminum-based adsorbents offer an attractive approach to effective, selective fluoride removal due to the thermodynamic stability of the aluminum-fluoride bond (Haynes, 2009). Activated alumina is ubiquitous in utility-scale and household-scale water treatment in high income countries and middle class communities throughout the world, and effectively removes fluoride provided pH and co-occurring ions are appropriately managed (Dahi and Chiang MaiThailand, 2000; Choi and Chen, 1979; Farrah et al., 1987). However, the cost of activated alumina, which stems largely from the energy-intensive process of purifying (Chen and Graedel, 2012a; Patterson, 1967) and modifying raw bauxite ores at temperatures exceeding 1000 °C (Chen and Graedel, 2012b), makes the materials economically unattainable to low-income populations.

Previous work in our group (Cherukumilli et al., 2017a) and by others has demonstrated that raw bauxite ore from a variety of locations (including Iran (Malakootian et al., 2014), Ghana (Buamah et al., 2013), India (Das et al., 2005), Malawi (Kayira et al., 2014; Sajidu et al., 2008), and Turkey (Dilek et al., 2013)) can be used to remove fluoride from drinking water at significantly lower costs than activated alumina. Bauxite ore is composed largely of aluminum hydroxides, along with significant or trace quantities of iron-, silicon-, and sometimes calcium- and titanium-oxides. Our previous research shows that when pH is controlled between 5.5 and 6.5, pulverized bauxite ore used as a dispersive batch adsorbent can reliably bring fluoride levels to below the WHO-MCL (1.5 mg F-/L) (Cherukumilli et al., 2017a). Bauxite that has been thermally activated at low temperatures (200–400 °C) has been further demonstrated to be a more effective fluoride adsorbent by Das and coworkers (Das et al., 2005), Peter (2009), and more recently by our group (Cherukumilli et al., 2018). Our work estimated that on a per-water-treated basis, raw bauxite costs roughly 23X less as a dispersive batch adsorbent for fluoride removal than activated alumina (Cherukumilli et al., 2017a), and that this cost is further reduced using thermally activated bauxite, accounting for increased bauxite treatment costs but reduced material transportation costs due to a lower required bauxite dose (Cherukumilli et al., 2018). Such a significant cost reduction clearly points to an opportunity to scale this research for the benefit of low-income communities.

One third of the globally reported cases of fluorosis occur in India (UNICEF, 1999), where the majority of states report regions with groundwater fluoride concentration in excess of the WHO MCL (Central Ground Water Board Ministry of Water Resources Government of India, 2010). The Nalgonda district in Telengana, India, where skeletal fluorosis is endemic, is relatively close (<500 km distant) to Visakhapatnam in Andhra Pradesh, India, from where the bauxite used in this study is mined. Due to the geographically proximate abundant supply of bauxite, it is one example of an appropriate location to pilot a safe drinking water project in this region using locally sourced bauxite. The research presented in this work helps to fulfill the technical needs of such a pilot project. However, because technical readiness is only one component of successfully launching a technology, it is important to present this research in the broader context of technology implementation. To achieve successful technology integration through community partnerships and business practices, this project aims to follow models and lessons learned from the transition from laboratory to field pilot of electrochemical arsenic remediation (ECAR) for arsenic removal in Dhapdhapi, India (Amrose et al., 2014, 2015).

The model that our team followed when scaling ECAR from lab to field included four key steps. First, the team conducted social surveys to get an understanding of the community's risk-perception of arsenic and evaluate their interest in having a treatment facility installed (Das et al., 2016). In parallel, through cost-analyses, the team confirmed that the technology could produce healthy water at a locally affordable price, ensuring that it would be financially viable (Roy, 2008). Third, input from the community was gathered from open meetings and interviews and consultation with key community opinion leaders to ensure that ECAR design and operation would be culturally appropriate (Amrose et al., 2014; Delaire et al., 2017). Finally, after construction and commissioning, the plant was thoroughly tested for over a year before water was distributed to the community. This gave the team adequate time to confirm that the treated water fully met the standards for drinking water even under varying operating conditions, which included seasonal changes and occasional operator neglect. In conclusion in order for a technology to be useful, sustainable, and be considered as potentially scalable it must be (1) desired by the community, (2) affordable, (3) culturally appropriate, (4) technically effective, and (5) robust in the relevant operating environment.

The success-to-date of the ECAR model will guide the design of a pilot fluoride remediation plant, and this paper focuses on point 4–technical effectiveness. As we proceed, it is crucial to understand when bauxite can be safely and effectively used to treat drinking water, and if there are any major technical limitations. Indeed, this is the point at which many promising water treatment discoveries die at the laboratory bench; while initial scientific results are promising, the resources are often lacking to uncover latent problems and elucidate their resolution to create a technology. The “valley of death” faced in introducing a technology to market is often discussed as a business challenge; however, the barrier imposed by the details necessary to make the leap from test tube to pilot plant are equally daunting. In the development engineering context in particular, resource constraints often mean that once a technology enters an initial pilot stage, the technology implementers may get only “one shot” in terms of community perception and trust; the technology must succeed the first time it is unveiled, or lack of additional funding and loss-of-trust will not allow for a second attempt as it often possible for stable, large companies targeting high-income markets. Thus, exploring in a scientifically rigorous manner the technical constraints that may threaten the success of a new technology allows the operation of the first pilot to remain well inside the margins of failure, which is one crucial aspect of technology adoption.

Within the context of technical effectiveness, major parameters that can impact all water treatment technologies include pH, co-occurring ions (i.e. ions that are not themselves a health concern, but may interfere with removal of hazardous contaminants), and the inadvertent release of hazardous chemicals from the materials used in the water treatment process. An additional concern that is specific to adsorbent media for water treatment is the reversibility of binding of a target contaminant to the adsorbent. This is important both for the possible regeneration of adsorbent media (thereby reducing waste), and for selection of appropriate treatment setups to prevent re-release of removed contaminants into the environment, aquifers, and even in treated water upon prolonged contact.

With regards to pH, the pH adsorption envelope for effective removal of fluoride by bauxite has been well-characterized (Habuda-Stanic et al., 2014; Cherukumilli et al., 2017a; Das et al., 2005; Sujana and Anand, 2011; Tomar and Kumar, 2013) with maximum adsorption typically between 5.5 and 6.5, similar to those for effective fluoride removal by activated alumina (Leyva-Ramos et al., 2008) and other aluminum oxides and hydroxides (Pommerenk and Schafran, 2005; Hingston et al., 1974; Telutli-Sequeira et al., 2012; Sujana et al., 1998). This suggests that working within these pH constraints is achievable at scale, and thus is not explored in depth in this paper. In some cases pH adjustment is not necessary because water pH falls within, or near enough to the optimal range, for effective fluoride removal. For situations where this is not the case, field-appropriate methods for acidifying groundwater pH via addition of acid or CO_2_ during batch adsorption tests with bauxite are explored in our recent work (Cherukumilli et al., 2018). This work recommends thermal activation of bauxite as a first step towards improved fluoride removal, but does find that these acidification methods are cost-effective when considered in the context of the corresponding reduced bauxite transportation costs from working in an optimal pH range. In particular, inexpensive, widely available technologies used by shopkeepers for carbonation of water may be appropriate for household- and community-scale water treatment.

In this work, we study three other factors relevant to the safe removal of fluoride from drinking water at scale: the effect of co-occurring ions on adsorption, the potential reversibility of adsorption and the potential leaching of metals into water during defluoridation with bauxite. With regards to co-occurring ions, we explore the impacts of phosphate and sulfate as potentially competitive co-occurring ions on the adsorption of fluoride onto both raw and thermally activated Vizag bauxite, building on our previous work (Cherukumilli et al., 2017b). We also test gibbsite, widely postulated to be the active adsorbent material in bauxite, in the presence of nitrate, silicic acid, phosphate and sulfate (Habuda-Stanic et al., 2014; Kayira et al., 2014; Sujana and Anand, 2011), to provide fundamental understanding in a simpler model system. Due to the lack of observed interference of nitrate and silicic acid in fluoride adsorption in gibbsite, these were not studied as competitors to fluoride adsorption on either raw or thermally activated bauxite.

We construct isotherms for the adsorption of fluoride in the absence and presence of these ions at varied concentrations, and fit these isotherms to widely used theoretical adsorption models. For those ions that significantly influence fluoride adsorption, we measure the final concentration in solution to provide insight into the mechanism of competition (i.e. whether the co-occurring ion is directly binding to the adsorbent). To understand reversibility of fluoride adsorption, we further construct forward and reverse adsorption isotherms, (i.e. starting with fluoride in either the solution or adsorbed on the surface). Finally, we apply the United States Environmental Protection Agency (US EPA)'s Toxicity Characteristic Leaching Procedure (TCLP Method 1311) (United States Environmental Protection Agency, 1992) to a sample of raw bauxite to determine whether measurable amounts of any contaminants of concern are released into product water. The extreme conditions required in the TCLP method allow a cautious overestimate of possible release of metals into water, meaning that a non-detect result provides a good buffer of confidence in the safety of the tested material for use in water treatment.

Combined, the results in this paper answer crucial questions for the practical application of bauxite as a low-cost adsorbent for fluoride remediation, helping to bridge the technical readiness gap to bring this technology to communities impacted by fluorosis.

### 1.1 Co-occurring ions

The impacts of co-occurring ions on fluoride adsorption on aluminum-based adsorbents have also been explored in a preliminary manner by other groups. Studies on adsorbents including alum sludge, alumina-coated magnetite nanoparticles, aluminum hydroxide, activated alumina, and bauxite consistently find that of all common anions in groundwater, phosphate concentrations (tested between 10 and 300 mg/L) have the greatest impact on fluoride adsorption (Sujana and Anand, 2011; Sujana et al., 1998; Gai et al., 2015; Chai et al., 2013; Tang et al., 2009). In addition, a study of high-activity aluminum hydroxide suggests that phosphate inhibits fluoride adsorption by binding to the surface of the aluminum hydroxide (Gai et al., 2015). These studies also consistently agree that nitrate has little to no effect on fluoride removal; the same is consistently found with chloride (Cherukumilli et al., 2017a; Sujana and Anand, 2011; Sujana et al., 1998; Gai et al., 2015; Chai et al., 2013; Tang et al., 2009).

Studies of sulfate as a co-occurring ion have more varied results. A study on activated alumina and activated bauxite concluded that sulfate had no effect on fluoride removal using either adsorbent (Choi and Chen, 1979). On the other hand, several studies on adsorbents such as an alumina/chitosan composite, ultrasonically prepared high activity aluminum hydroxide, and alum sludge, the waste material of alum manufacture, find that sulfate concentrations as low as 10 mg/L reduce fluoride adsorption, though in each case sulfate competes to a lesser extent than phosphate (Gai et al., 2015; Viswanathan and Meenakshi, 2010). In addition, many studies only look at the effects of sulfate at concentrations of up to 200 mg/L, which is lower than what may be found in many groundwater sources (the US EPA secondary drinking water standard is 250 mg/L (United States Environmental Protection Agency, ), and many groundwater matrices contain more than double this amount) (World Health Organization, 2004a). Although their study covers much lower concentrations of competing ions than this work, (5–25 mg/L) Sujana and Anand find similar results for fluoride adsorption on raw bauxite from Orissa, India, demonstrating that fluoride adsorption is slightly impacted by sulfate and dramatically impacted by phosphate (Sujana and Anand, 2011). While bauxite samples vary by region, this adsorbent is quite similar to the one used in our work (Cherukumilli et al., 2017a).

There are fewer results on the effect of silicate (whether in its anionic form, or in its predominant form in groundwater, as a neutral species, silicic acid, below pH 9.84) on fluoride adsorption, and these results are also mixed. On alum sludge, the effect of silicate is close to that of phosphate and is significantly greater than that of sulfate (Sujana et al., 1998). Choi and Chen also find that silicate reduces fluoride adsorption onto activated alumina, but find that it has no effect on adsorption onto bauxite (Choi and Chen, 1979). They hypothesize that this difference is because of the presence of large amounts of silicate as an impurity in the bauxite used in their study. Existing reports in the competitive-ion literature generally fail to control solution pH throughout the experiment, making it difficult to decouple the reduction in fluoride adsorption caused by the presence of co-occurring anions from the effect of variation in pH.

Controlling solution pH is particularly important for water matrices containing silicate and phosphate because these ions act as competing buffers and dramatically influence pH (Ripin and Evans, 2005; Alexander et al., 1954). Examining the effects of co-occurring anions at concentrations equal to or exceeding those that commonly occur allows for a better understanding of the limitations of this technology in the field.

### 1.2 Fluoride adsorption reversibility

The reversible binding of fluoride to adsorbent media has been explored for several adsorbents, but to-date there are no reports in the literature characterizing reversibility of fluoride binding to bauxite at circumneutral to slightly acidic pH, where adsorption of fluoride onto bauxite is most effective. The majority of literature around reversibility of binding examines only the desorption of fluoride from adsorbents at elevated pH where fluoride desorption occurs readily (Habuda-Stanic et al., 2014; Das et al., 2005; Hingston et al., 1974; Jinadasa et al., 1988; Mohapatra et al., 2004), although fluoride adsorption is demonstrated to be reversible from goethite at pH 4.5 (Hingston et al., 1974). The authors of this earlier study note that adsorbents exchange at the adsorbate surface more readily in a solution with high ionic strength of non-competing ions. The most rigorous isotherm studies are performed by Leyva-Ramos and coworkers, who demonstrate that fluoride adsorbs reversibly to bone char (hydroxyapatite) at pH 7 and 12 (Medellin-Castillo et al., 2007), and that, by contrast, adsorption of fluoride to activated alumina is not thermodynamically reversible at pH 5, but is reversible at pH 12 (Leyva-Ramos et al., 2008). We expect the behavior of bauxite to more closely correspond to the latter of these two results since it is an aluminum-based adsorbent. The latter result is also consistent with what is observed by Gai and coworkers (Gai et al., 2015), who study desorption as a function of pH for ultrasonically prepared Al(OH)_3_, and find that fluoride binding is reversible only at a pH of 12 or greater.

Understanding the reversibility of fluoride adsorption is important for determining appropriate water treatment plant setups; for instance, if passing water with a low concentration of fluoride over fluoride-saturated media can cause fluoride to desorb back into solution to concentrations in excess of the WHO MCL, the use of a packed bed might be an inappropriate choice without suitable precautions. Similarly in this reversible adsorption scenario, a tube settler could be used following batch treatment and with water that has consistent levels of fluoride, but would be constrained in its utility if water from multiple sources with varied fluoride concentrations were treated in the same facility because fluoride could desorb into water with lower concentrations. As a counterpoint to this, if desorption occurs at circumneutral pH, regeneration of the adsorbent media may be possible without the use of strongly basic solutions.

### 1.3 Safety of adsorbent material

Concerns around the inadvertent release of hazardous materials into water during treatment particularly manifest with the use of adsorbent materials. Because adsorbents are solid media with a high surface area, they have potential to leach their constituent minerals into the water. In the case of mineral adsorbents, their constituents may have a well-characterized “safe” level in drinking water (e.g., the WHO MCL for Arsenic is 0.01 mg/L (World Health Organization, 2004b); the EPA secondary drinking water limit for aluminum is 0.05–0.2 mg/L) (United States Environmental Protection Agency, ). Preliminary work in our group has indicated that under acidic conditions, the raw bauxite used in this study (from Vishakapatnam, India) may release levels of aluminum in excess of the EPA secondary MCL and thermally activated bauxite may release levels of manganese in excess of the EPA secondary MCL (0.05 mg/L) (Cherukumilli et al., 2017b); these metals will need to be mitigated in technology development. Additionally, “stress-testing” the system under longer contact times and chemically harsher conditions is necessary to understand the limitations on the safe operating conditions of the adsorbent materials. While secondary standards do not indicate acute concerns with the safety of water, they do indicate degradation of aesthetic quality (color, taste, turbidity) and therefore potential challenges in user acceptance of the treated water. Reliably providing safe, aesthetically appealing water is crucial to the adoption of a water treatment technology.

## 2 Materials, methods, and approaches

### 2.1 Adsorbent materials

Bauxite was collected from a mine in Visakhapatnam, Andhra Pradesh, India. Gibbsite was received from Alcoa. After oven drying each sample at 100 °C for 24 h to remove moisture, 5 g of bauxite was milled for 15 min and 5 g of gibbsite for 1 h in a stainless steel milling jar of a shaker ball mill (SPEX 8000 or SPEX 8000M) to generate micron sized powders. The milling time for the gibbsite was chosen from among several possible time intervals because it provided material with the surface area (by BET nitrogen adsorption, Gibbsite: 15.1 ± 2.1 m^2^/g) that was closest to that previously reported for Bauxite: 11.0 ± 3.0 m^2^/g (Cherukumilli et al., 2017b). Some of the powdered bauxite was then heated at 300 °C for 4 h in a muffle furnace (Fisher Scientific, IsoTemp) to produce “thermally activated bauxite” according to the procedure of Cherukumilli et al. (2017b) Activated bauxite was demonstrated in our previous work to have significantly higher adsorption capacity for fluoride as compared to raw bauxite, as well as a higher surface area by BET, Activated Bauxite: 173 ± 25 m^2^/g (Cherukumilli et al., 2017b). These values are consistent with trends in surface area of aluminum oxide and hydroxide species reported in the literature (Das et al., 2005; Fleming and Goodboy, 1990).

### 2.2 Materials characterization

Bulk elemental composition of the bauxite was measured by energy dispersive X-Ray fluorescence spectroscopy and specific surface area of the milled gibbsite, bauxite, and activated bauxite was measured using Multipoint Brunauer-Emmett-Teller (BET) as in Cherukumilli et al. (2017a)

### 2.3 Isotherm adsorption experiments

All experiments were conducted at room temperature (22–25 °C).

*Standards:* Calibration curves were constructed using standards with fluoride concentrations of 0, 1, 2, 5, 10, 20, 40, 80, and 120 mg/L in a groundwater matrix prepared using 50 mM MES buffer adjusted to a pH of 6.0 ± 0.2. Standards were prepared with a constant initial ionic strength of 100 mM, within the typical concentration range of total dissolved solids in groundwater, using sodium chloride as an “indifferent electrolyte” to balance ionic strength because chloride is known not to influence fluoride adsorption on bauxite (Cherukumilli et al., 2017a). In cases where a co-occurring ion was added, the concentration of sodium chloride was correspondingly reduced to maintain an ionic strength of 100 mM. Each standard was diluted with an equal volume of Total Ionic Strength Adjustment Buffer (TISABII) to complex any free aluminum and iron, and free-fluoride (F^−^) was measured using a fluoride ion-selective electrode (Mettler Toledo perfectION). Standards with the appropriate groundwater matrix composition were used to construct separate calibration curves for each set of experiments, although it was noted that the presence of co-occurring ions in the standards did not influence reading from the ion-selective electrode.

*Samples:* The groundwater matrix was spiked with 0, 5, 10, 20, 40, 60, 80, 100 mg/L of fluoride (F^−^) as sodium fluoride, and prepared using 50 mM MES buffer adjusted to a pH of 6.0 ± 0.2, consistent with the adsorption envelope of fluoride on bauxite and other aluminum-based adsorbents that show high adsorption capacity at this pH (Cherukumilli et al., 2017a; Sujana and Anand, 2011; Gai et al., 2015). The pH was measured to increase by < 0.2 pH units for all samples throughout the experiment. Samples were prepared with a constant initial ionic strength of 100 mM, using sodium chloride as an “indifferent electrolyte” to balance ionic strength (Cherukumilli et al., 2017a). In cases where a co-occurring ion was added, the concentration of sodium chloride was correspondingly reduced to maintain an ionic strength of 100 mM. For each experiment, 10 mL samples of this spiked simple synthetic groundwater matrix were placed in 15 mL polypropylene centrifuge tubes and 1 g/L of adsorbent (gibbsite, bauxite or activated bauxite) was added based on preliminary experiments that indicated this dose removes a modest amount of fluoride over the 0–100 ppm fluoride concentration range, providing meaningful measurements and isotherms for comparison.

The tubes were affixed to a rotisserie tube rotator and the suspensions were mixed for 24 h to ensure equilibrium was reached. Upon completion of each adsorption experiment, 2.8 mL aliquots from each slurry were collected with a syringe and filtered using a 0.2 μm filter before analysis. Filtered aliquots were then mixed with equal volumes of Total Ionic Strength Adjustment Buffer (TISABII) to complex any free aluminum and iron, and free-fluoride (F^−^) was measured using a fluoride ion-selective electrode (Mettler Toledo perfectION). The adsorption density was determined by subtracting the remaining dissolved fluoride concentration from the initial dissolved fluoride concentration and dividing by the mass of adsorbent. pH was measured before and after addition of adsorbent, and again after 24 h of mixing.

All experiments were completed in triplicate or greater. X-axis error bars in adsorption isotherm graphs represent the standard deviation in the measured equilibrium fluoride concentrations in all experiments. Y-axis error bars represent standard error in adsorption density, as calculated using standard error propagation formulas (Navidi, 2011), based on the estimated error in the initial fluoride concentration due to pipette errors (provided by manufacturer), the standard deviation in equilibrium fluoride concentration, and the assumed error in adsorbent mass due to error associated with the use of an analytical balance. Error calculated for concentrations determined by ion chromatography is the standard deviation in the measured ion concentrations over three experiments. Significance testing was done for points at 10 ppm initial fluoride concentration, because this represents the high end of typical groundwater fluoride concentrations found in areas suffering endemic fluorosis (World Health Organization, 2006). The significance of the difference between the equilibrium fluoride concentrations in samples with and without the co-occurring anion in question was tested using a one-tailed, two sample *t*-test assuming unequal variance. The null hypothesis was that there is no difference in the mean equilibrium fluoride concentration between the two samples, and the alternative hypothesis was that the equilibrium fluoride concentration in the presence of the co-occurring anion was greater than that in the absence of the anion in question. The null hypothesis was rejected in favor of the alternative hypothesis if p < 0.05.

#### 2.3.1 Competition experiments

Common groundwater concentrations for the anions studied in this work (phosphate, sulfate, silicic acid, and nitrate), along with MCL values when they exist, are listed in Table 1.

**Table 1:** Common Groundwater Concentrations and MCL values for Anions in Groundwater.

Ion (Form at pH = 6)	Typical Concentration (mg/L, mM)	Maximum Contaminant Level (mg/L, mM) (United States Environmental Protection Agency, )	Concentrations Tested in this Study (mM)
Phosphate (H_2_PO_4_^−^)	Below detection limit (Handa, 1975) to parts per thousand levels with contamination (Handa, 1975)	none	1, 5, 25 mM
Sulfate (SO_4_^2−^)	0–230 mg/L (0–2.2 mM), much higher (parts per thousand) with contamination (World Health Organization, 2004a)	250 mg/L (2.4 mM) *(secondary)*	1, 5, 25 mM
Nitrate (NO_3_^−^)	<10 mg/L, up to 1500 mg/L (<0.16–25 mM) with agricultural contamination (World Health Organization, 2011)	10 mg/L (0.16 mM) *(primary)*	25 mM
Silicic acid (H_4_SiO_4_)	pH – dependent; solubility limit ∼0.2 ppm (1.5 mM) at pH 6	none	1 mM
Chloride (Cl^−^)	wide range, including brackish waters	250 mg/L (7.1 mM) *(secondary)*	N/A

Samples containing 0–100 mg/L fluoride were prepared containing 1, 5, or 25 mM sulfate (as Na_2_SO_4_), 1, 5, or 25 mM phosphate (as NaH_2_PO_4_), 25 mM nitrate (as NaNO_3_), or 1 mM silicate (as NaSiO_3_·5H_2_O, maximum concentration due to solubility limitations of silicic acid) (Alexander et al., 1954). Competition by sulfate and phosphate on gibbsite was tested at the noted concentrations to provide a range between extremely high contaminant concentration (25 mM, intentionally higher than those typically reported in groundwater) and likely concentrations in “good” water (∼1 mM), with 5 mM denoting a middle ground and realistic concentration in water that would otherwise be accepted as potable in many low-resource contexts. In the case of sulfate, 5 mM is roughly double the US EPA secondary drinking water standard (See Table 1). (United States Environmental Protection Agency, ).

Data for fluoride adsorption on pure gibbsite were collected at a range of co-occurring ion concentrations. Due to the lack of clear correlation between ion concentration and degree of competition (see Results and Discussion), we focused on a single concentration representative of likely sulfate and phosphate concentrations found in the field for adsorption studies on bauxite and thermally activated bauxite. Possible values can vary widely for phosphate because it often comes from anthropogenic contamination, and so we used drinking water regulations for sulfate to determine a reasonable value for testing both ions, based on similar perception of salinity and taste quality that would arise from sulfate and phosphate. Sulfate is regulated by the US EPA as a secondary contaminant with a concentration limit of 250 mg/L, or roughly 2.4 mM (United States Environmental Protection Agency, ); this concentration is not enforced and serves as a guideline, and many water sources can have higher concentrations (World Health Organization, 2004a). We selected 5 mM as an appropriate representative concentration, higher than what would ideally be consumed, but within a range to which populations with limited choice in safe drinking water supply are likely habituated.

Due to the lack of observed interference of nitrate and silicic acid in fluoride adsorption in gibbsite, these were not studied as competitors to fluoride adsorption on either raw or thermally activated bauxite.

#### 2.3.2 Ion chromatography

For samples containing sulfate and phosphate, additional aliquots of supernatant were collected after 24 h of mixing (at the same time as those collected for measurement of fluoride concentration) and filtered through a 0.2 μm filter. These additional samples were then diluted appropriately for analysis by Ion Chromatography (Dionex ICs 1100, Anion Mode, using Dionex Seven Anion Standard I diluted between 1 and 100 times to construct a calibration curve). For comparison, the concentrations of sulfate and phosphate were measured in samples that had not been exposed to any adsorbent material, and compared to the concentrations of those exposed to adsorbent.

#### 2.3.3 Fluoride adsorption reversibility experiments

The reversibility of the adsorption of fluoride on gibbsite, bauxite, and thermally activated bauxite at pH 6 was determined by performing adsorption experiments with samples containing 0–100 mg/L fluoride. As in competition experiments (Section 2.3.1) 10 mL samples were mixed on a rotisserie tube rotator for 22 h. They were then allowed to sit for 2 h (for a total contact time of 24 h) to maximize the settling of suspended adsorbents to the bottom of the centrifuge tube. 9.5 mL of supernatant were then carefully withdrawn from the tube via syringe, passing “backwards” through a 0.2 μm filter in order to capture any suspended adsorbent. This left 0.5 mL of solution and the majority of adsorbent in the bottom of the tube. 2.8 mL of the filtered supernatant was recovered and mixed with an equal volume of TISAB II for measurement of fluoride concentration. 9.5 mL of fluoride-free simple synthetic groundwater (50 mM MES buffer adjusted to pH 6, and sodium chloride to bring the total ionic strength to 100 mM) was then passed “forwards” through the same filter in three aliquots to wash any captured adsorbent back into the centrifuge tube, returning the sample volume to 10 mL. The samples were then affixed to a rotisserie tube rotator and the suspensions mixed for 72 h. Thus, the final fluoride content of the sample is the sum of 5% of that measured at the end of adsorption, plus whatever is subsequently desorbed. Upon completion of each desorption experiment, an additional 2.8 mL aliquot from each slurry was collected in a syringe, filtered using a 0.2 μm filter and combined with an equal volume of TISAB II for analysis. If fluoride adsorption is reversible, then the fluoride would desorb back into solution, and the desorption isotherm obtained from this second set of samples would be expected to be found on the same line as the adsorption isotherm.

#### 2.3.4 Fitting of adsorption isotherms

Isotherms were fitted using ISOFIT software (Matott, 2007), which uses a combination of particle swarm optimization and Levenberg–Marquardt nonlinear regression to minimize the weighted sum of squared error. The average across the triplicate experiments, as well as the corresponding adsorption density measurement errors (the calculation of which is detailed in Section 2.3) were input into the ISOFIT software. Each observation was assigned weighting inversely proportional to its associated measurement error (Hill, 1998). All isotherms supported by ISOFIT were fitted.

The goodness of fit was evaluated using the correlation between measured and fitted observations, the standard deviation of regression, and the corrected Akaike Information Criterion (AICc) (Hurvich and Tsai, 1994), as computed by ISOFIT. The AICc is a measure that allows one to compare and rank multiple models and select which best approximates the “true” process (Symonds and Moussalli, 2011). Since the AICc only derives meaning in comparison with the AICc values of other models, the correlation coefficient and standard deviation were additionally used to evaluate overall quality of fit.

### 2.4 United States Environmental Protection Agency Toxicity Characteristic Leaching Procedure (US EPA TCLP)

A sample of raw bauxite was submitted for analysis to Curtis & Tompkins, in Berkeley, California. The standard EPA TCLP Leaching Procedure, Method 1311^44^ was applied to the sample, and metals in the leachate were analyzed via EPA Methods and 7470 (mercury) (United States Environmental Protection Agency, 1994a) and 6020 (all other metals) (United States Environmental Protection Agency, 1994b). The US Environmental Protection Agency's Toxicity Characteristic Leaching Procedure (TCLP), Method 1311 (United States Environmental Protection Agency, 1992), is intended to simulate conditions of what might be released if a material was deposited in a landfill and then exposed to acidic or alkaline runoff. Thus it is a more extreme condition than is likely to be encountered in water treatment, with a more drastic pH range, higher mass of bauxite per volume of water, and longer exposure times.

## 3 Results and discussion

### 3.1 Adsorption isotherms for fluoride as influenced by the presence of Co-occurring ions

The effects of several co-occurring ions on fluoride adsorption density onto gibbsite, bauxite, and thermally activated bauxite were determined by assembling isotherms for a range of initial fluoride concentrations from 0 to 100 mg/L. The following series of graphs show adsorption density of fluoride as a function of equilibrium fluoride concentration in solution on gibbsite, raw bauxite, and bauxite thermally activated at 300 °C.

**Fig. 1:**
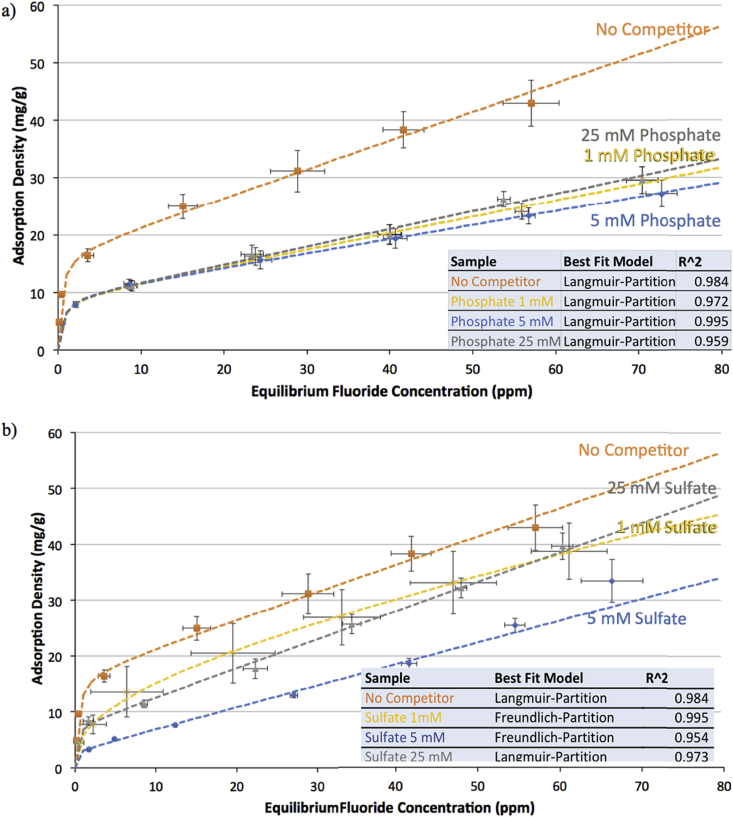

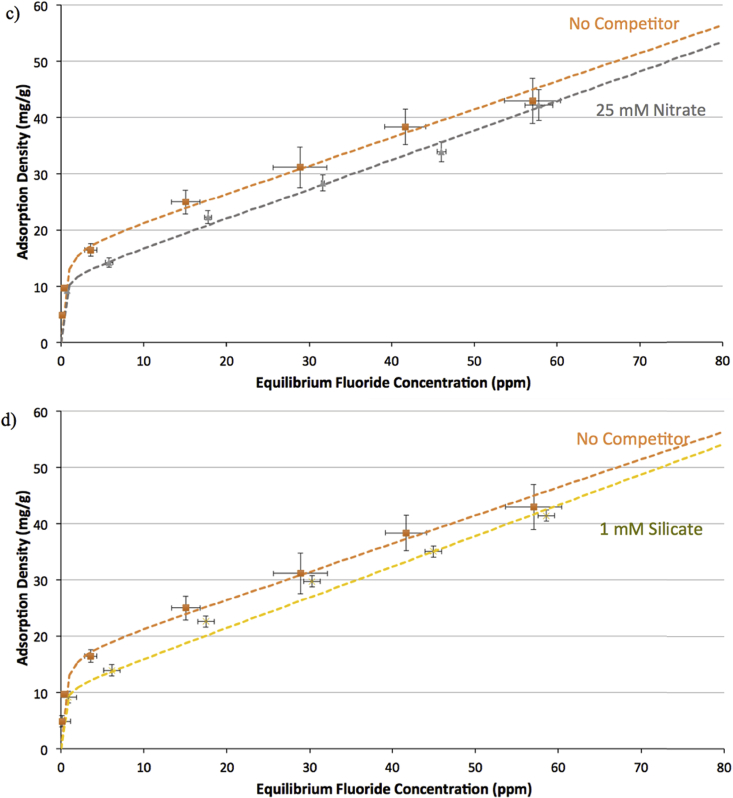
Adsorption isotherms for fluoride on gibbsite at pH = 6.0 ± 0.2 and ionic strength of 100 mM, for a) phosphate (1, 5, and 25 mM), b) sulfate (1, 5, and 25 mM), c) nitrate (25 mM), and d) silicate (as silicic acid) (1 mM). Data are shown for adsorption in the absence of competing ions (orange) and in the presence of competing ions (yellow = 1 mM, blue = 5 mM, grey = 25 mM). Error bars on all data points represent one standard error above and below the mean for three or more trials. Dashed lines indicate the best fit isotherm model determined by Isofit – isotherm parameters are found in the Supporting Information.

Fig. 1 shows adsorption isotherms of fluoride on gibbsite, comparing the isotherm generated in the absence of co-occurring ions to those generated in the presence of phosphate (at 1,5, and 25 mM), sulfate (at 1,5, and 25 mM), silicate (silicic acid, at 1 mM), and nitrate (at 25 mM).

Fig. 1 a) indicates that the presence of phosphate dramatically reduces the adsorption of fluoride on gibbsite. For an initial fluoride concentration of 10 ppm (which is within the range commonly found in contaminated drinking water sources), the reduction in fluoride removal is approximately five-fold in the presence of phosphate; the final fluoride concentrations with and without phosphate are respectively ∼0.4 ppm and ∼2.1 ppm. Significance testing also suggests that the reduction in fluoride removal at 10 ppm initial fluoride concentration in the presence of phosphate is statistically significant. The threshold for saturation of the effect of the phosphate ion is very low, below 1 mM (the lowest concentration tested). Above this concentration, variation in the amount of phosphate does not significantly impact fluoride adsorption to a statistically significant degree. This is consistent with observations by Sujana and Anand (2011), who find that any amount of co-occurring phosphate up to ∼0.2 mM has approximately the same negative effect on fluoride adsorption. Although it is quite common for fluoride to occur in groundwater with no detectable phosphate (Handa, 1975), the finding that phosphate impacts fluoride removal has practical implications because phosphate is often introduced to water supplies by contamination with agricultural runoff (of surface water) or infiltration (into groundwater – although the mobility of phosphate into groundwater is lower than that of nitrate) (British Geological Survey, 2000). This is a particular risk in rural areas where adequate remediation technologies are often needed, and highlights the importance both of protecting groundwater sources and of testing for secondary contaminants that may be introduced in the process of water extraction.

Fig. 4 b) shows that, like phosphate, the presence of sulfate reduces fluoride adsorption on gibbsite, but to a variable extent. The reduction in fluoride removal from an initial concentration of 10 ppm fluoride varies from 4-fold to 12-fold. The correlation between sulfate concentration and fluoride adsorption is inconsistent, with the presence of 5 mM sulfate resulting in a statistically significant reduction in adsorption capacity compared to either 25 mM or 1 mM sulfate. At 5 mM and 25 mM sulfate, the reduction in fluoride removal compared to that in the absence of sulfate is statistically significant, while at 1 mM sulfate, the effect is not significant. It is not clear what causes this variability, but Sujana and Anand observe similar results (Sujana and Anand, 2011).

Because both phosphate and sulfate were shown to significantly impact fluoride adsorption on gibbsite, the residual concentrations of these two ions after treatment of synthetic groundwater with gibbsite was also analyzed. Only in the case of 1 mM sulfate solution, did final concentrations of ions after exposure to gibbsite differ significantly from the initial concentrations (See Supporting Information). This is in contrast with previous observations by Gai and coworkers, who find that ultrasonically prepared aluminum hydroxide removes a significant quantity of phosphate from solution, and also find that phosphate has a much greater negative impact on fluoride adsorption than sulfate or any other ions studied (Gai et al., 2015).

From Fig. 1 c) and d), it is evident that neither nitrate nor silicic acid significantly influence the adsorption of fluoride on gibbsite. For nitrate this is consistent with numerous other reports (Sujana and Anand, 2011; Gai et al., 2015), which generally show that both nitrate and chloride are spectator ions in fluoride adsorption or have very limited effect compared to other ions.

Literature reports of the effect of silicate on fluoride adsorption are sparse; studies that include silicate as a co-occurring ion generally do not account for pH and for the fact that silicate is neutral at pH below 9 and thus has limited solubility. However, there is literature precedent to suggest that silicate itself binds to gibbsite (Adu-Wusu and Wilcox, 1991; Jepson et al., 1976). Given that the first pKa of Silicic acid (Si(OH)_4_) is 9.04,^52^ and therefore addition of NaSiO_3_·5H_2_O into an unbuffered solution will significantly alter the pH, the existing literature provides limited opportunities to compare our observation of no significant competition between fluoride and silicate for binding to gibbsite.

Using the software ISOFIT (Matott, 2007), we modeled the adsorption isotherms of fluoride binding to gibbsite in the presence and absence of co-occurring ions. On gibbsite in the absence of co-occurring ions, the model with the best fit is Langmuir-Partition, a dual mode isotherm that incorporates both Langmuir and Linear terms. In the presence of phosphate at all concentrations and of sulfate at 25 mM, Langmuir-Partition remains the best fit for the fluoride adsorption isotherm. The reduction in both the adsorption capacity and affinity parameters ( $K_{f}$ and $\;n_{f}$) of the model in the presence of phosphate affirms that phosphate inhibits fluoride adsorption. However, for sulfate at 1 mM and 5 mM, the Freundlich-Partition model is the best fit, though the Langmuir-Partition model remains a good fit for sulfate at 1 mM as well. Our results are similar to those in the literature on isotherms for describing adsorption of fluoride onto aluminum-based adsorbents. Both Langmuir- and Freundlich-type isotherms are often found to be suitable for describing adsorption, though most examples in the literature consider only the Langmuir and Freundlich models (Sujana et al., 1998; Chai et al., 2013; Viswanathan and Meenakshi, 2010). Sujana and Anand, for example, found Langmuir to best describe adsorption on raw bauxite from Orissa, India (Sujana and Anand, 2011), while Cherukumilli et al. found the Freundlich isotherm to be the best model for adsorption on raw bauxite from several geographies (Cherukumilli et al., 2017a). In all cases, these models are based on ideal systems; particularly at low concentrations the Langmuir term dominates in any mixed Langmuir models. The variations in best-fitting model are not sufficiently drastic for us to conclude any fundamental differences in adsorption mechanism in our non-ideal system, but the fitting to conventional isotherms supports that surface adsorption is generally responsible for fluoride removal.

**Fig. 2:**
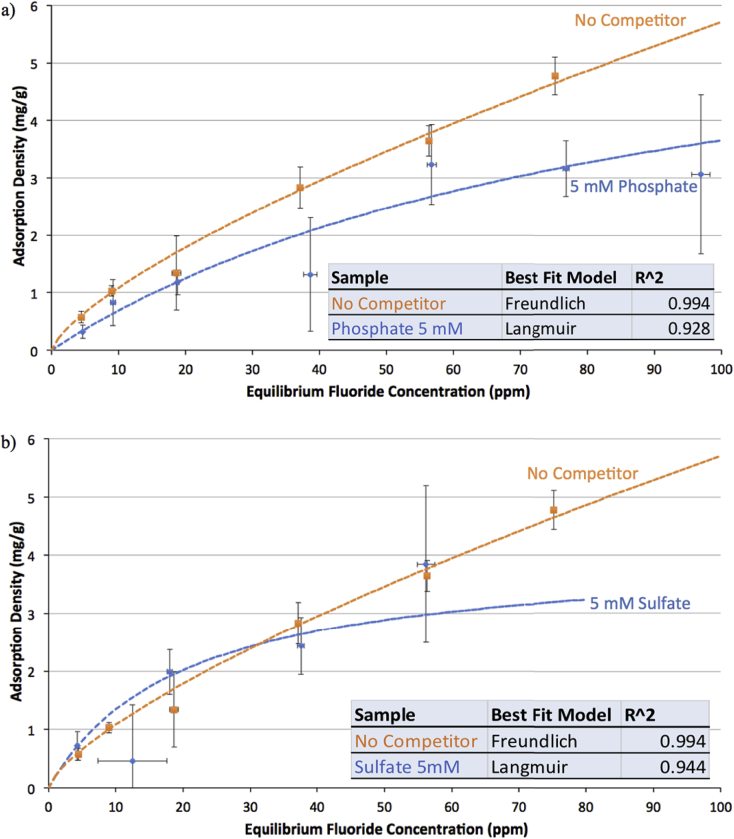
Adsorption isotherms for fluoride on raw bauxite at pH = 6.0 ± 0.2 and ionic strength of 100 mM, in the absence of competing ions (orange) and in the presence of a) phosphate (5 mM) and b) sulfate (5 mM) (both blue). Error bars on all data points represent one standard error above and below the mean for three trials. Dashed lines indicate the best fit isotherm model determined by Isofit – isotherm parameters are found in the Supporting Information.

**Fig. 3:**
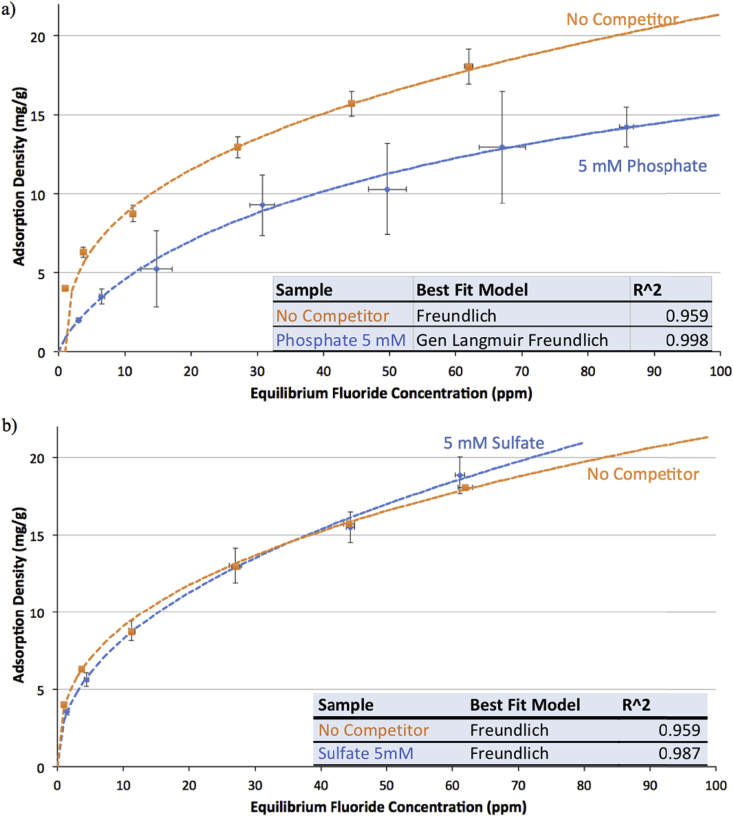
Adsorption isotherms for fluoride on bauxite thermally activated at 300 °C, at pH = 6.0 ± 0.2 and ionic strength of 100 mM, in the absence of competing ions (orange) and in the presence of a) phosphate (5 mM) and b) sulfate (5 mM) (both blue). Error bars on all data points represent one standard error above and below the mean for three trials. Dashed lines indicate the best fit isotherm model determined by Isofit – isotherm parameters are found in the Supporting Information.

Fig. 2 shows adsorption isotherms of fluoride on raw bauxite, comparing the isotherm generated in the absence of co-occurring ions to those generated in the presence of phosphate (at 5 mM) and sulfate (at 5 mM). Similarly, Fig. 3 shows adsorption isotherms of fluoride on bauxite thermally activated at 300 °C in the absence of co-occurring ions and in the presence of phosphate (at 5 mM) and sulfate (at 5 mM).

From Fig. 2, Fig. 3 we see that the presence of 5 mM phosphate leads to a reduction in fluoride adsorption on both raw (at high fluoride concentrations only) and thermally activated bauxite (at all concentrations of fluoride, with roughly a two-fold reduction in adsorption at 10 ppm fluoride). These results are consistent with the gibbsite experiments and findings in the existing literature (Sujana and Anand, 2011; Gai et al., 2015). By contrast, the presence of sulfate reduces fluoride adsorption on gibbsite (a 4- to 12-fold reduction depending on sulfate concentration), but appears to have no effect on fluoride adsorption on thermally activated bauxite (Fig. 3 b). The Langmuir model fitted to the adsorption data in the presence of sulfate suggests that the presence of sulfate may reduce adsorption onto bauxite at fluoride concentrations above of the range normally encountered in real groundwater, although the individual data points show no significant effect (Fig. 2 b). Ion chromatography shows significant removal of both sulfate and phosphate by activated bauxite, but no significant removal of either ion by raw bauxite, likely due to the significantly lower surface area of the raw bauxite (See Supporting Information).

On both raw bauxite and thermally activated bauxite, the Freundlich isotherm provides the best fit when there is no competitor. The intrinsic adsorption capacity calculated is lower than what has been observed in other studies on fluoride adsorption onto bauxite (Chen and Graedel, 2012b), though this may be partly due to the lower surface area of the bauxite used in this study compared to other studies. The closer fit to a non-ideal isotherm, which is built around variation in the affinity of the adsorbent to different binding sites suggests that there is greater heterogeneity in these adsorbents than in gibbsite, on which adsorption in the absence of a competitor was best described by a Langmuir-type isotherm. This may be due to the presence of other materials in the bauxite (minerals of iron, silicon, titanium, and calcium) (Cherukumilli et al., 2017a), that serve as adsorption sites. For raw bauxite, in the presence of sulfate and phosphate at 5 mM, a Langmuir isotherm provides the best fit. On activated bauxite in the presence of phosphate, the best fit model in ISOFIT is a Generalized Langmuir-Freundlich isotherm, while the presence of sulfate has no effect on the shape of the isotherm, and a Freundlich isotherm is still the best fit.

Overall, the results of these experiments indicate that the specific kinds of co-occurring ions in real groundwater will influence the adsorbent dose, and thus be relevant to successful fluoride remediation. Silicic acid and nitrate did not interfere with fluoride adsorption at circumneutral pH on gibbsite. Sulfate, however, has been shown to significantly impact the adsorption of fluoride on gibbsite at sulfate concentrations ranging from 5 to 25 mM, causing a 4- to 12-fold reduction in fluoride adsorption from initial fluoride concentrations of 10 ppm. While sulfate did not significantly impact fluoride adsorption on raw or thermally activated bauxite at typical groundwater fluoride concentrations, the results with model adsorbent gibbsite, the variability at higher groundwater fluoride concentrations, and literature precedent (Sujana and Anand, 2011; Tang et al., 2009) all suggest that the impacts of sulfate should be carefully monitored and mitigated in pilot plant studies. Phosphate consistently and significantly impacts fluoride adsorption for all three adsorbents studied at all concentrations tested (1–25 mM), reducing fluoride adsorption 5-fold on gibbsite and 2-fold on thermally activated bauxite (both from initial fluoride concentrations of 10 ppm). On a systems level, contamination of groundwater with phosphate can often be avoided. These results highlight the importance of land management practices that protect groundwater aquifers from contamination by leaching of agricultural runoff.

### 3.2 Hysteresis: isotherms for adsorption and desorption of fluoride

**Fig. 4:**
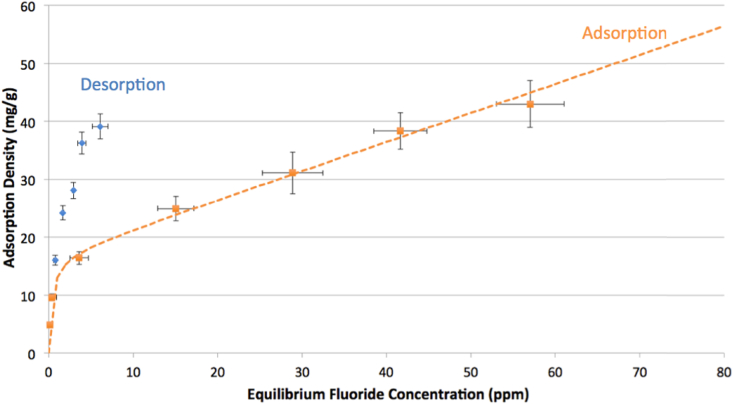
Adsorption and desorption isotherms for fluoride on gibbsite at pH = 6.0 ± 0.2 and ionic strength of 100 mM. Error bars represent one standard error above and below the mean for three or more trials. Dashed line indicates the best fit isotherm model determined by Isofit for forward adsorption – isotherm parameters are found in the Supporting Information.

**Fig. 5:**
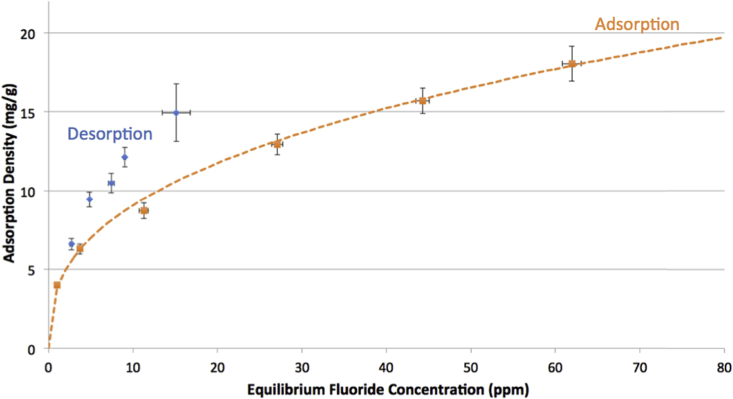
Adsorption and desorption isotherms for fluoride on activated bauxite at pH = 6.0 ± 0.2 and ionic strength of 100 mM. Error bars represent one standard error above and below the mean for three or more trials. Dashed line indicates the best fit isotherm model determined by Isofit for forward adsorption – isotherm parameters are found in the Supporting Information.

**Fig. 6:**
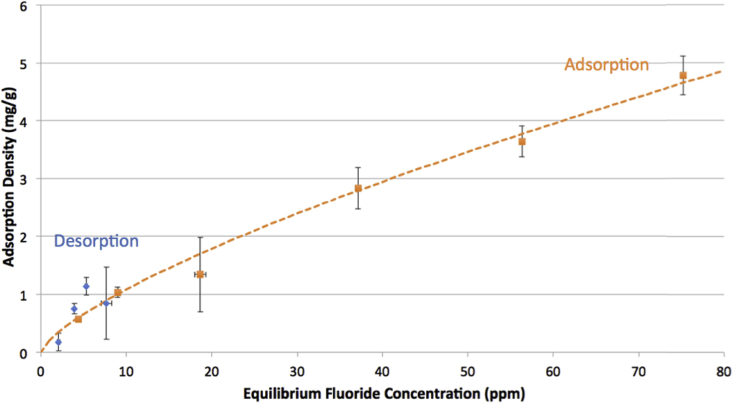
Adsorption and desorption isotherms for fluoride on raw bauxite at pH = 6.0 ± 0.2 and ionic strength of 100 mM. Error bars represent one standard error above and below the mean for three or more trials. Dashed line indicates the best fit isotherm model determined by Isofit for forward adsorption – isotherm parameters are found in the Supporting Information.

Graphs Fig. 4, Fig. 5, Fig. 6 show adsorption and desorption curves for gibbsite, bauxite, and thermally activated bauxite, respectively, all at pH 6. As discussed in Section 3.2, the adsorption isotherm on gibbsite fits a Langmuir Partition model, while those on raw and activated bauxite both fit a Freundlich model. If fluoride adsorption were reversible, desorption data would fall along the same line and fit the same isotherm. However, desorption data from raw or activated bauxite does not fit any model currently in the ISOFIT software, and desorption from gibbsite fits very poorly to linear and Langmuir-Partition models. It is clear that adsorption is not thermodynamically reversible for gibbsite or activated bauxite; this is consistent with literature on fluoride adsorption to aluminum-based adsorbents (Leyva-Ramos et al., 2008; Gai et al., 2015). For gibbsite, the amount of fluoride released back into solution is less than 1.5 ppm for all samples with original fluoride concentrations of 60 ppm or less, suggesting that there is no realistic situation where gibbsite used to treat drinking water could re-contaminate treated water at pH 6. Similarly, adsorption of fluoride onto thermally activated bauxite is not thermodynamically reversible; activated bauxite initially exposed to 20 ppm fluoride (with an adsorption density of 9 mg fluoride/g activated bauxite) releases only 2.1 ppm of fluoride back into solution. 20 ppm is far above normal levels of fluoride in water, and so a lower adsorption density and lower level of release of fluoride would be anticipated for real groundwater.

For raw bauxite, it is more difficult to assess whether adsorption is effectively reversible because fluoride adsorption densities are so low. The data for desorption equilibria appears to fall along the adsorption isotherm, although the lack of fit to a model supports the assertion that the behavior of fluoride on raw bauxite is consistent with other aluminum adsorbents. The amount released from a sample initially exposed to 20 ppm fluoride (with an adsorption density of 0.6 mg fluoride/g bauxite) is 1.2 ppm. Preliminary experiments at higher bauxite loadings (4 and 10 g bauxite/L water rather than the reported 1 g/L) indicated the same trend, with the low adsorption capacity of raw bauxite limiting the ability to conclusively assess the thermodynamic reversibility of this process.

### 3.3 Safety of adsorbent material

It is essential to regularly test drinking water for hazardous contaminants pre- and post-fluoride remediation at all sites where a new technology is implemented. To supplement this, a standard, certified testing method can demonstrate whether contaminants of concern are likely to be released into the water. This testing provides either a reassurance of safety or an opportunity to proactively mitigate potential contaminants. The US Environmental Protection Agency's Toxicity Characteristic Leaching Procedure (TCLP), Method 1311 (United States Environmental Protection Agency, 1992), was chosen as a more extreme condition than typical in water treatment to provide a margin of safety in this analysis. Full results of the TCLP and subsequent analysis of the leachate are found in the supporting information. Notable in the results is the detection of Arsenic (0.017 mg/L, reporting limit 0.0075; WHO MCL 0.01 mg/L) and Manganese (2.0 mg/L, reporting limit 0.032; EPA secondary MCL 0.05 mg/L). Manganese was also detected as a contaminant in our previous work (Cherukumilli et al., 2017b) and while aluminum was not detected in this leaching experiment, it should be noted that the detection limit of the instrument was 0.5 mg/L, while the EPA secondary MCL is 0.05–0.2 mg/L and our previous work detected aluminum using a more sensitive instrument (Cherukumilli et al., 2017b). Gai and coworkers find that leaching of aluminum from gibbsite is strongly temperature dependent (Gai et al., 2015), this may also be true for bauxite and be an important factor to consider for field implementation in warm climates. Notable and encouraging is the fact that none of cadmium, chromium, lead, or mercury, four highly toxic metals, were detected in this sample.

The presence of arsenic in TCLP leachate at a level above the WHO MCL is of potential concern, although, as noted, the conditions of the TCLP are more extreme than those of standard water treatment. Arsenic was not detected in water samples after treatment with bauxite in our previous work (detection limit 0.1 ppb by ICP-MS) (EAG Laboratories, ), so it is unlikely that under actual treatment conditions, toxic levels of arsenic will leach into treated water (Cherukumilli et al., 2018). Overall, the TCLP results are encouraging to continue further development of technologies based on adsorption of fluoride by bauxite, and serve as a reminder of the importance of ongoing testing of actual water samples to ensure that the drinking water being provided to communities is safe.

### 3.4 Application and limitations in future field pilot context

A major challenge of using bauxite, activated alumina, and any other adsorbents shown to be effective for the removal of fluoride from drinking water is the pH dependence of the adsorbents, with maximum performance achieved in a pH range 5.5–6.5 (Habuda-Stanic et al., 2014; Cherukumilli et al., 2017a; Das et al., 2005; Sujana and Anand, 2011; Tomar and Kumar, 2013). In this study, we controlled the pH to 6.0, based on previous findings demonstrating that using carbon dioxide or hydrochloric acid to control the pH of water should be financially feasible (Cherukumilli et al., 2018). Technical feasibility in a field pilot remains to be demonstrated; it is worth noting that the presence of calcium carbonate in Vizag bauxite makes it one of the more challenging bauxite sources to use; bauxite in our previous work sourced from Guinea, Ghana, and the United States resulted in equilibrium pH values between 6.2 and 6.6 without addition of acid (Cherukumilli et al., 2017a).

Notwithstanding changes in the overall effectiveness of bauxite, the results of the present study with regards to impacts of co-occurring ions should hold within higher pH ranges that are still within a reasonable range for drinking water. Sulfate will not undergo a change in protonation state, and while the second pKa of phosphoric acid is 7.21, the transition from a monoanion to a dianion will not likely significantly impact interactions (Ripin and Evans, 2005).

In on our previous work, the adsorption isotherms and envelopes were very similar for a range of bauxite ores, sourced from India, Guinea, Ghana, and the United States, when water pH was controlled to ∼6, both in the absence of co-occurring ions and in a simulated complex groundwater mixture (Cherukumilli et al., 2017a). This suggests that the results of the present work can be applicable beyond the Nalgonda area, in geographical regions impacted by fluorosis but with different locally-available bauxite sources. Dosing of bauxite to achieve fluoride removal down to the WHO MCL or 1.5 mg/L will have to be validated with local groundwater regardless, but the similar properties of diverse bauxite ores suggest that competition with co-occurring ions will have a similar profile, and preliminary analysis of the groundwater matrix in an area will support initial estimates of required bauxite doses and additional materials cost of implementing water treatment.

## 4 Conclusion and prospectus

When transitioning from laboratory to field with an innovation targeted at communities in developing countries, understanding the technical risks is essential to defining tolerances for safe, effective design parameters for the new technology. In the present work, we explore the effects of co-occurring ions, reversibility of fluoride adsorption, and potential for leaching of hazardous metals when using raw and thermally activated bauxite as an adsorbent to remove fluoride from drinking water, with bauxite sourced from Visakhapatnam, Andhra Pradesh, India and using synthetic gibbsite as a simple model material.

When synthetic groundwater is buffered at pH 6, we find that co-occurring phosphate negatively impacts the adsorption capacity of fluoride onto of all three adsorbents: 5-fold reduction in adsorption capacity on gibbsite at 10 ppm fluoride, 2-fold reduction in adsorption capacity on activated bauxite at 10 ppm fluoride; significant effects are observed only at high concentrations of fluoride on activated bauxite, and so may not have noticeable effects in the field, depending on context. Sulfate negatively impacts the adsorption capacity of gibbsite (by a factor of 4- to 12-fold from an initial fluoride concentration of 10 ppm). Thus, the potential presence of phosphate and sulfate in groundwater needs to be accounted for to reliably design a system that removes sufficient fluoride from drinking water. Silicate (as silicic acid) and nitrate do not affect fluoride adsorption capacity in this study.

Further, we find that adsorption of fluoride onto gibbsite and thermally activated bauxite is not thermodynamically reversible (the adsorption capacity of raw bauxite is too low to conclude about fluoride adsorption reversibility via the methods used, although lack of model fit suggests the same conclusion); this indicates that contact of saturated adsorbent with safe drinking water during a processing, settling, or other separation step is not likely to impact water safety and is therefore not a design constraint, although monitoring of water post-treatment is still important, and will remain so until all steps in the technical process are fully understood, including the parametric limits on their effective performance.

Finally, we find that leachate from raw bauxite obtained via a United States EPA leaching protocol shows levels of arsenic above the WHO-MCL; the extreme nature of this protocol means that arsenic is likely not a cause for concern when treating water with bauxite. The material does not have hazardous levels of any other primary drinking water contaminants, although previous studies indicate that aluminum leaching from bauxite may be a concern. As a precaution, levels of both aluminum and arsenic should be monitored in drinking water treated with raw or thermally activated bauxite.

Incorporating an understanding of all of these technical constraints into the design of a technology for fluoride remediation will increase the likelihood of success of early prototypes, accelerating the path to using bauxite as a low-cost adsorbent to provide fluoride-safe drinking water to communities throughout the developing world.
