# Supplementary material for: Addressing technical barriers for reliable, safe removal of fluoride from drinking water using minimally processed bauxite ores
Source: Dev Eng. 2018;3:175–87. doi: 10.1016/j.deveng.2018.06.002 (PMC6277820; doi:10.1016/j.deveng.2018.06.002)
Supplement: Multimedia component 1 [file mmc1.docx]

Supporting Information for:

**Addressing technical barriers for reliable, safe removal of fluoride from drinking water using minimally processed bauxite ores**

Heather L. Buckley,^1*^ Nusrat J. Molla,^2^ Katya C. Cherukumilli,^2^ Kathryn S. Boden,^2^ Ashok J. Gadgil^1,2^

^1^Energy Technology Area, Lawrence Berkeley National Laboratory, Berkeley, CA, USA, 94720

^2^Civil and Environmental Engineering, University of California Berkeley, Berkeley, CA, USA, 94720

*Corresponding author, email: hlbuckley@lbl.gov

Table of contents:

1. **Description of Fluoride Adsorption Isotherm Data Tables for Competition Experiments S3**
   1. **Table S1: Fluoride Adsorption Density vs. Equilibrium Fluoride Concentration for Competing Ions on Gibbsite**
      1. **S1.a. Phosphate**
      2. **S1.b. Sulfate**
      3. **S1.c. Nitrate**
      4. **S1.d. Silicate**
   2. **Table S2: Fluoride Adsorption Density vs. Equilibrium Fluoride Concentration for Competing Ions on Bauxite and Activated Bauxite**
      1. **S2.a. Phosphate – Bauxite**
      2. **S2.b. Sulfate – Bauxite**
      3. **S2.c. Phosphate – Activated Bauxite**
      4. **S2.d. Sulfate – Activated Bauxite**
2. **Description of Ion Chromatography Tables S3**
   1. **Table S3: Residual Competing Ions in Gibbsite Adsorption Experiments**
   2. **Table S4: Residual Competing Ions in Bauxite and Thermally Activated Bauxite Adsorption Experiments**
3. **Description of Fluoride Adsorption Isotherm Data Tables for Hysteresis Experiments S4**
   1. **Table S5: Fluoride Adsorption Density vs. Equilibrium Fluoride Concentration for Forward and Reverse Adsorption**
      1. **S5.a Gibbsite**
      2. **S5.b Raw Bauxite,**
      3. **S5c. Thermally Activated Bauxite**
4. **Description of Curtis & Tompkins TCLP Report S4**
5. **References S4**

**A. Description of Fluoride Adsorption Isotherm Data Tables for Competition Experiments**

**Table S1: Fluoride Adsorption Density vs. Equilibrium Fluoride Concentration for Competing Ions on Gibbsite**

These tables show the measured fluoride concentrations (based on a calibration curve of known standard solutions) and calculated fluoride adsorption densities onto gibbsite. Both raw data and averaged data with error are presented for fluoride solutions prepared as described in the Methods section, containing either no competing ions or a) phosphate, b) sulfate, c) nitrate or d) silicate. Additionally, these spreadsheets display the results of the Isofit^1^ analysis of adsorption isotherm model fitting as described in the Methods section.

File Name: “S1. Gibbsite Competition.xlsx”

**Table S2: Fluoride Adsorption Density vs. Equilibrium Fluoride Concentration for Competing Ions on Bauxite and Thermally Activated Bauxite**

These tables show the measured fluoride concentrations (based on a calibration curve of known standard solutions) and calculated fluoride adsorption densities onto raw bauxite and thermally activated bauxite. Both raw data and averaged data with error are presented for fluoride solutions prepared as described in the Methods section, containing either no competing ions or a) phosphate, or b) sulfate. Additionally, these spreadsheets display the results of the Isofit analysis of adsorption isotherm model fitting as described in the Methods section.

File Name: “S2. BauxActBaux Competition.xlsx”

**B. Description of Ion Chromatography Tables**

**Table S3: Residual Competing Ions in Gibbsite Adsorption Experiments**

These tables show the residual co-occurring ion concentrations measured by Ion Chromatography (based on a calibration curve of known standard solutions) after exposure of solutions to gibbsite, as compared to solutions that have not been exposed to adsorbent. Both raw data and averaged data with error are presented for solutions prepared as described in the Methods section; phosphate and sulfate are measured.

File Name: “S3. IC_Gibbsite.xlsx”

**Table S4: Residual Competing Ions in Bauxite and Thermally Activated Bauxite Adsorption Experiments**

These tables show the residual co-occurring ion concentrations measured by Ion Chromatography (based on a calibration curve of known standard solutions) after exposure of solutions to bauxite or thermally activated bauxite, as compared to solutions that have not been exposed to adsorbent. Both raw data and averaged data with error are presented for solutions prepared as described in the Methods section; phosphate and sulfate are measured.

File Name: “S4. IC_BauxiteActBaux.xlsx”

**C. Description of Fluoride Adsorption Isotherm Data Tables for Hysteresis Experiments**

**Table S5: Fluoride Adsorption Density vs. Equilibrium Fluoride Concentration for Forward and Reverse Adsorption on Gibbsite, Raw Bauxite, and Activated Bauxite**

This table shows the measured fluoride concentrations (based on a calibration curve of known standard solutions) and calculated fluoride adsorption densities onto gibbsite, raw bauxite, and activated bauxite.. Both raw data and averaged data with error are presented for fluoride solutions prepared as described in the Methods section for both forward and reverse adsorption. Additionally, these spreadsheets display the results of the Isofit analysis of adsorption isotherm model fitting as described in the Methods section.

File Name: “S5. Hysteresis.xlsx”

**D. Description of Curtis & Tompkins TCLP Report**

This report contains the certified analysis conducted by Curtis & Tompkins on a sample of raw bauxite. The sample was processed using EPA TCLP Leaching Procedure, Method 1311,^2^ and metals were analyzed via EPA Methods 6020^3^ and 7470.^4^ The report narrative notes situations where the method blank detected metals at non-zero concentrations. These baseline concentrations are accounted for in analysis conducted in this paper.

File Name: “S6. C&T TCLP Raw Bauxite.pdf”

**E. References**

(1) Matott, L. S. Isofit Version 1.2, 2007.

(2) United States Environmental Protection Agency. *Method 1311: Toxicity Characteristic Leaching Procedure*; 1992.

(3) United States Environmental Protection Agency. *Method 6020: Inductively Coupled Plasma - Mass Spectrometry*; 1994.

(4) United States Environmental Protection Agency. *Method 7470A Mercury in Liquid Waste (Manual Cold-Vapor Technique)*; 1994.
