# Supplementary material for: Addressing technical barriers for reliable, safe removal of fluoride from drinking water using minimally processed bauxite ores
Source: Dev Eng. 2018;3:175–87. doi: 10.1016/j.deveng.2018.06.002 (PMC6277820; doi:10.1016/j.deveng.2018.06.002)
Supplement: Multimedia component 7 [file mmc7.pdf]

Curtis & Tompkins, Ltd.

Target Analyte List Metals

Lab #:	289582	Project#:	STANDARD
Client:	Heather Buckley		
Field ID:	INDIA BAUXITE RAW	Sampled:	05/03/17
Lab ID:	289582-001	Received:	05/03/17
Matrix:	TCLP Leachate	Analyzed:	06/07/17
Units:	mg/L		

Analyte	Result	RL	Diln Fac	Batch#	Prepared	Prep	Analysis
Aluminum	ND	0.50	50.00	248537	06/06/17	EPA 3010A	EPA 6020
Antimony	ND	0.0050	50.00	248537	06/06/17	EPA 3010A	EPA 6020
Arsenic	0.017 b	0.0075	50.00	248537	06/06/17	EPA 3010A	EPA 6020
Barium	0.24 b	0.0089	50.00	248537	06/06/17	EPA 3010A	EPA 6020
Beryllium	ND	0.0050	50.00	248537	06/06/17	EPA 3010A	EPA 6020
Cadmium	ND	0.0099	50.00	248537	06/06/17	EPA 3010A	EPA 6020
Calcium	620	3.3	100.0	248537	06/06/17	EPA 3010A	EPA 6020
Chromium	ND	0.0050	50.00	248537	06/06/17	EPA 3010A	EPA 6020
Cobalt	0.018	0.0050	50.00	248537	06/06/17	EPA 3010A	EPA 6020
Copper	ND	0.041	50.00	248537	06/06/17	EPA 3010A	EPA 6020
Iron	2.6	0.87	50.00	248537	06/06/17	EPA 3010A	EPA 6020
Lead	ND	0.0050	50.00	248537	06/06/17	EPA 3010A	EPA 6020
Magnesium	7.6	0.50	50.00	248537	06/06/17	EPA 3010A	EPA 6020
Manganese	2.0	0.032	50.00	248537	06/06/17	EPA 3010A	EPA 6020
Mercury	ND	0.0010	1.000	248558	06/07/17	METHOD	EPA 7470A
Molybdenum	ND	0.010	50.00	248537	06/06/17	EPA 3010A	EPA 6020
Nickel	ND	0.0075	50.00	248537	06/06/17	EPA 3010A	EPA 6020
Potassium	ND	2.5	50.00	248537	06/06/17	EPA 3010A	EPA 6020
Selenium	ND	0.0076	50.00	248537	06/06/17	EPA 3010A	EPA 6020
Silver	ND	0.0050	50.00	248537	06/06/17	EPA 3010A	EPA 6020
Sodium	1,400 b	5.0	100.0	248537	06/06/17	EPA 3010A	EPA 6020
Thallium	ND	0.0028	50.00	248537	06/06/17	EPA 3010A	EPA 6020
Vanadium	ND	0.050	100.0	248537	06/06/17	EPA 3010A	EPA 6020
Zinc	ND	0.23	50.00	248537	06/06/17	EPA 3010A	EPA 6020

b= See narrative
ND= Not Detected
RL= Reporting Limit

Curtis & Tompkins, Ltd.

Batch QC Report

Target Analyte List Metals

Lab #:	289582	Prep:	EPA 3010A
Client:	Heather Buckley	Analysis:	EPA 6020
Project#:	STANDARD		
Type:	BLANK	Diln Fac:	25.00
Lab ID:	QC888725	Batch#:	248537
Matrix:	TCLP Leachate	Prepared:	06/06/17
Units:	mg/L	Analyzed:	06/07/17

Analyte	Result	RL
Aluminum	ND	0.25
Antimony	ND	0.0025
Arsenic	0.0059 b	0.0038
Barium	0.088 b	0.0025
Beryllium	ND	0.0025
Cadmium	ND	0.0049
Calcium	1.1 b	0.83
Chromium	0.0026 b	0.0025
Cobalt	ND	0.0025
Copper	ND	0.021
Iron	ND	0.43
Lead	ND	0.0025
Magnesium	ND	0.25
Manganese	ND	0.016
Molybdenum	ND	0.0050
Nickel	ND	0.0038
Potassium	4.2 b	0.25
Selenium	ND	0.0038
Silver	ND	0.0025
Sodium	1,500 >LR b	1.3
Thallium	ND	0.0014
Vanadium	ND b	0.0038
Zinc	ND	0.11

b= See narrative
ND= Not Detected
RL= Reporting Limit
>LR= Response exceeds instrument's linear range

Curtis & Tompkins, Ltd.

Batch QC Report

Target Analyte List Metals

Lab #:	289582	Prep:	EPA 3010A
Client:	Heather Buckley	Analysis:	EPA 6020
Project#:	STANDARD		
Type:	LCS	Diln Fac:	25.00
Lab ID:	QC888726	Batch#:	248537
Matrix:	TCLP Leachate	Prepared:	06/06/17
Units:	mg/L	Analyzed:	06/07/17

Analyte	Spiked	Result	%REC	Limits
Aluminum	10.00	10.33	103	80-134
Antimony	0.1000	0.09673	97	80-120
Arsenic	0.1000	0.1006	101	80-120
Barium	0.1000	0.1015	101	80-120
Beryllium	0.1000	0.09553	96	80-120
Cadmium	0.1000	0.1019	102	80-120
Calcium	10.00	10.05	100	80-120
Chromium	0.1000	0.09675	97	80-120
Cobalt	0.1000	0.09950	100	80-120
Copper	0.1000	0.09838	98	80-120
Iron	10.00	10.76	108	80-120
Lead	0.1000	0.1047	105	80-120
Magnesium	10.00	9.923	99	80-120
Manganese	0.1000	0.09478	95	80-120
Molybdenum	0.1000	0.09863	99	80-120
Nickel	0.1000	0.08728	87	80-120
Potassium	10.00	10.92 b	109	80-120
Selenium	0.1000	0.1023	102	80-120
Silver	0.1000	0.09985	100	80-120
Sodium	10.00	11.52	115	80-120
Thallium	0.05000	0.04833	97	80-120
Vanadium	0.1000	0.09200 b	92	80-120
Zinc	0.1000	0.08395	84	80-120

b= See narrative

Curtis & Tompkins, Ltd.

Batch QC Report

Target Analyte List Metals

Lab #:	289582	Prep:	METHOD
Client:	Heather Buckley	Analysis:	EPA 7470A
Project#:	STANDARD		
Analyte:	Mercury	Batch#:	248558
Matrix:	Water	Prepared:	06/07/17
Units:	mg/L	Analyzed:	06/07/17
Diln Fac:	1.000		

Type	Lab ID	Spiked	Result	%REC	Limits	RPD	Lim
BS	QC888802	0.002500	0.002414	97	80-120		
BSD	QC888803	0.002500	0.002378	95	80-120	2	20

RPD= Relative Percent Difference

Curtis & Tompkins, Ltd.

Batch QC Report

Target Analyte List Metals

Lab #:	289582	Prep:	METHOD
Client:	Heather Buckley	Analysis:	EPA 7470A
Project#:	STANDARD		
Analyte:	Mercury	Diln Fac:	1.000
Type:	BLANK	Batch#:	248558
Lab ID:	QC888806	Prepared:	06/07/17
Matrix:	TCLP Leachate	Analyzed:	06/07/17
Units:	mg/L		

Result	RL
ND	0.0010

ND= Not Detected
RL= Reporting Limit

Curtis & Tompkins, Ltd.

CASE NARRATIVE

Laboratory number: **289582**
Client: **Heather Buckley**
Request Date: **06/05/17**
Samples Received: **05/03/17**

This data package contains sample and QC results for one solid sample, requested for the above referenced project on 06/05/17. The sample was received cold and intact.

Metals (EPA 6020 and EPA 7470A):
Low response was observed for vanadium in the ICV analyzed 06/07/17 13:10; affected data was qualified with "b". Potassium and vanadium were detected at or above the RL in the ICB analyzed 06/07/17 13:27; affected data was qualified with "b". Potassium and vanadium were detected at or above the RL in the CCB analyzed 06/07/17 15:40; affected data was qualified with "b". Response exceeding the instrument's linear range was observed for sodium in the method blank for batch 248537; affected data was qualified with "b". A number of analytes were detected above the RL in the method blank for batch 248537. No other analytical problems were encountered.
